# Successful laparoscopic resection for gastric duplication cyst: a case report

**DOI:** 10.1186/s13256-019-2129-1

**Published:** 2019-07-19

**Authors:** Hideki Izumi, Hisamichi Yoshii, Rin Abe, Masaya Mukai, Eiji Nomura, Hiroyuki Ito, Tomoko Sugiyama, Takuma Tajiri, Hiroyasu Makuuchi

**Affiliations:** 1 Department of Gastrointestinal Surgery, Tokai University Hachioji Hospital, 1838 Ishikawa, Hachioji, Tokyo, 192-0032 Japan; 2 Department of Internal Medicine, Tokai University Hachioji Hospital, 1838 Ishikawa, Hachioji, Tokyo, 192-0032 Japan; 3 Department of Pathology, Tokai University Hachioji Hospital, 1838 Ishikawa, Hachioji, Tokyo, 192-0032 Japan; 4 Department of Diagnostic Radiology, Tokai University Hachioji Hospital, 1838 Ishikawa, Hachioji, Tokyo, 192-0032 Japan

**Keywords:** Gastric duplication cyst, Laparoscopic surgery, Gastric mucosa, Laparoscopy

## Abstract

**Background:**

Gastric duplication is a relatively rare congenital malformation, accounting for approximately 2.9–3.8% of gastrointestinal duplications. Gastric duplication cyst is a congenital anomaly that is rarely observed in adults. Accurate diagnosis of these cysts before resection is difficult. In this report, we describe a patient with gastric duplication cysts that were treated by laparoscopic resection.

**Case presentation:**

A 46-year-old Japanese woman was referred to our institution because a cystic lesion in the pancreatic tail was detected by ultrasonography during a health examination. The lesion had a clearly defined boundary of approximately 40 mm. A thick cystic lesion of the septum was observed in the pancreatic tail, but invasion into the stomach wall was not recognized on a computed tomographic scan. Endoscopic ultrasonography revealed that the tumor appeared smooth with a marginal edge, which was characterized by echo with high homogeneity, and the presence of viscous mucus was suspected. The preoperative diagnosis of mucinous cystic neoplasm was the reason for laparoscopic tumor resection. The resected specimen was a smooth surface tumor, and it was full of mucus. Histopathological study revealed that the mucosa was covered with crypt epithelium, muscularis mucosae, intrinsic muscularis, and serosa, and the wall of the tumor had a structure very similar to that of the stomach wall. The mucosa was partially drained by intrinsic gastric glands, but most of them were denucleated. No pancreatic tissue was present, and the tumor had no continuity with the spleen. These findings indicated a diagnosis of gastric duplication cyst with no continuity with the stomach wall.

**Conclusions:**

In our experience, it is difficult to differentiate gastric duplication cyst from mucinous cystic neoplasm before laparoscopic resection. Events such as infection, bleeding, perforation, ulceration, fistula formation, obstruction, and compression have been linked to gastric duplication cysts, and malignant transformation of these cysts has been reported. Therefore, we suggest that resection should be the first treatment option for gastric duplication cysts.

## Background

Gastric duplication cyst (GDC) is a congenital anomaly that is rarely observed in adults [1]. In approximately 50% of patients with GDCs, the cysts are found within the first year after birth with symptoms such as vomiting, abdominal pain, and weight loss, and more than 70% of reported cases have involved patients younger than 12 years [1]. In adults, most cases are incidentally discovered on radiological examination or gastric endoscopy [2].

Accurate diagnosis of these cysts before resection is difficult. Differential diagnoses are varied, including gastrointestinal stromal tumors, neuroendocrine tumors, pancreatic heterotopia, pancreatic pseudocysts, and neurogenic tumors [3].

GDCs are extremely difficult to diagnose preoperatively. We describe a case of a patient with laparoscopically resected GDCs, focusing on diagnostic and therapeutic methods. We initially misdiagnosed our patient as having mucinous cystic neoplasms (MCNs) and performed laparoscopic resection.

## Case presentation

Our patient was an otherwise healthy 46-year-old Japanese woman who was referred to our institution because a cystic lesion in the pancreatic tail was detected by ultrasonography during a health examination. Her past medical history and family medical history were unremarkable. She was not taking any medication. She did not have a smoking habit; however, she occasionally drank alcohol. Blood tests revealed no abnormality. Levels of tumor markers were not elevated (Table 1). She had no physical abnormalities at admission.

**Table 1:** Laboratory data on admission

WBC	5.4 × 10^3^	/ul
RBC	4.02 × 10^6^	/ul
Hb	12.4	g/dl
Ht	37.3	%
PLT	22.9 × 10^4^	/ul
BUN	14	mg/dl
Cr	0.62	mg/dl
Na	140	mEq/L
K	4.1	mEq/L
Cl	104	mEq/L
Ca	9.7	mg/dl
CRP	0.031	mg/dl
Alb	4.4	g/dl
CK	75	IU/L
GOT	21	IU/L
GPT	22	IU/L
ALP	182	IU/L
γ-GTP	19	IU/L
T-Bil	0.8	mg/dl
AMY	101	IU/L
CEA	3.2	ng/ml
CA19–9	1.0	U/ml

*WBC* white blood cell, *RBC* red blood cell, *Hb* hemoglobin, *Ht* hematocrit, *Plt* platelet, *BUN* blood urea nitrogen, *Cr* creatinine, *Na* sodium, *K* potassium, *Cl* cholride, *Ca* calcium, *CRP* C-reactive protein, *Alb* albumin, *CK* Creatine Kinase, *GOT* Glutamic Oxaloacetic Transaminase, *GPT* Glutamic Pyruvic Transaminase, *ALP* Alkaline Phosphatase, *γ-GTP* γ-glutamyl transpeptidase, *T-Bil* total bilirubin, *AMY* amylase, *CEA* Carcinoembryonic antigen, *CA19-9* Carbohydrate antigen 19-9

**Fig. 1:**
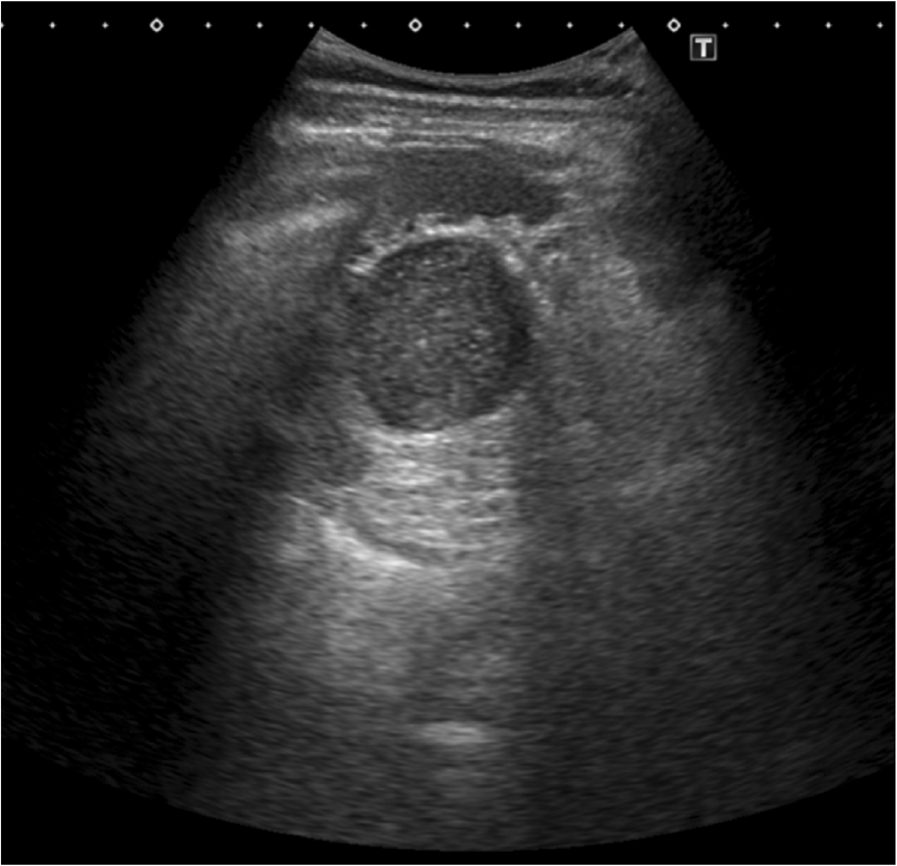
Abdominal ultrasound revealing a cystic lesion with a clearly defined boundary of approximately 40 mm in the pancreatic tail

**Fig. 2:**
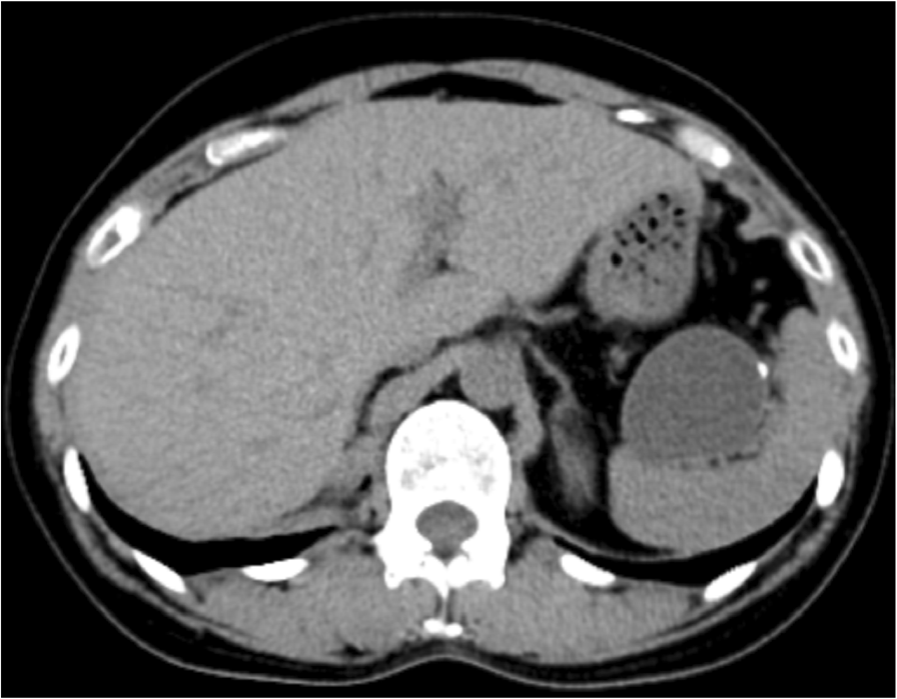
A thick cystic lesion of the septum is visible in the pancreatic tail, but computed tomographic scan shows no invasion into the stomach wall

**Fig. 3:**
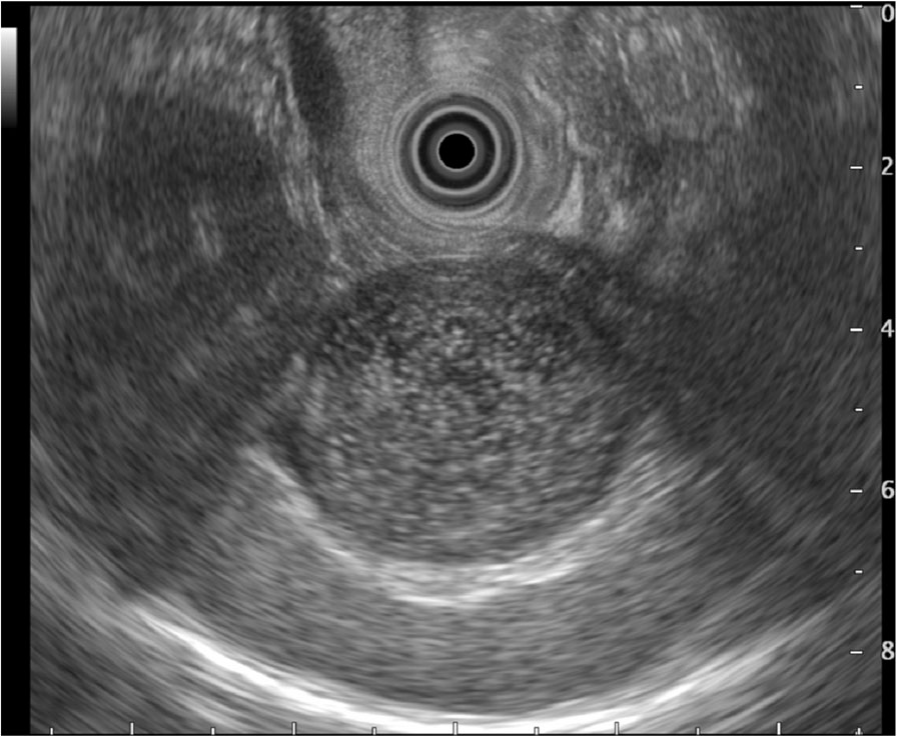
Endoscopic ultrasound showing the tumor, which appears smooth with a marginal edge, characterized by echo with high homogeneity, and the presence of viscous mucus was suspected

Abdominal ultrasonography (Fig. 1) revealed a thick cystic lesion of the septum with a clearly defined boundary of approximately 40 mm in the pancreatic tail; however, computed tomography revealed no invasion into the stomach wall (Fig. 2). Upper gastrointestinal endoscopy showed no obvious abnormality. Endoscopic ultrasonography (EUS) (Fig. 3) revealed that the tumor appeared smooth with a marginal edge and was characterized by echo with high homogeneity, and the presence of viscous mucus was suspected. The preoperative diagnosis was mucinous cystic neoplasm, and surgery was performed accordingly.

**Fig. 4:**
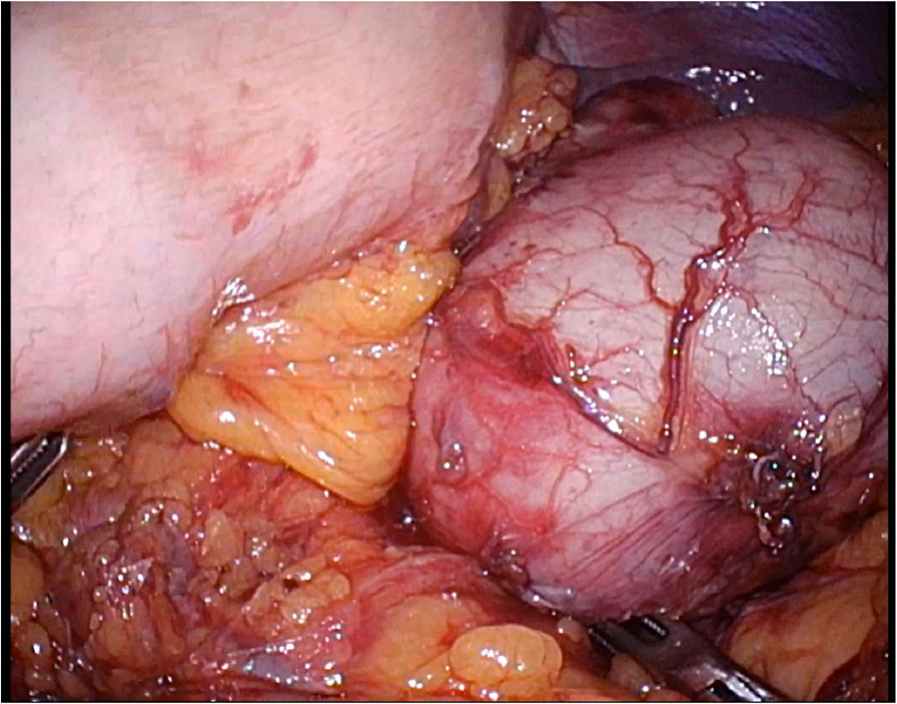
A soft tumor, whose surface was smooth, like the serosa of the stomach wall, was found in the pancreatic tail during laparoscopic surgery

**Fig. 5:**
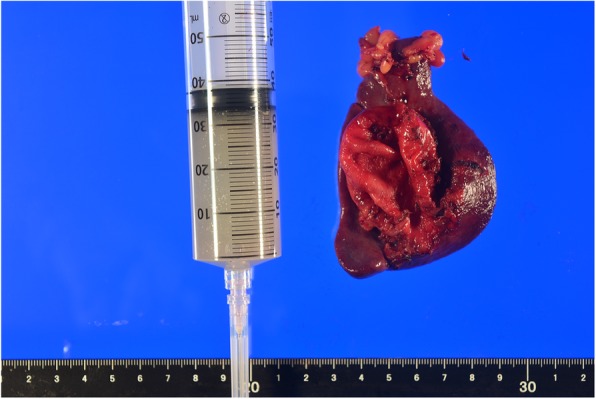
The resected specimen was a smooth surface tumor, and its contents consisted of mucus

During laparoscopic surgery, a soft tumor whose surface was smooth, like the serosa of the stomach wall, was found in the pancreatic tail (Fig. 4). There was no continuity between the tumor and stomach wall, and no adhesion was observed. When the tumor was peeled off the pancreatic tail, we determined that the tumor did not arise from the pancreas. Peeling the tumor off the splenic hilum was difficult because the adhesions between the two were strong; therefore, we excised the spleen along with the tumor. The cyst was retrieved in a bag and transected 4 cm above the pubic bone. The operative time was 129 min, and the bleeding volume was 50 ml. The resected specimen was a smooth surface tumor, and it comprised mucus (Fig. 5).

**Fig. 6:**
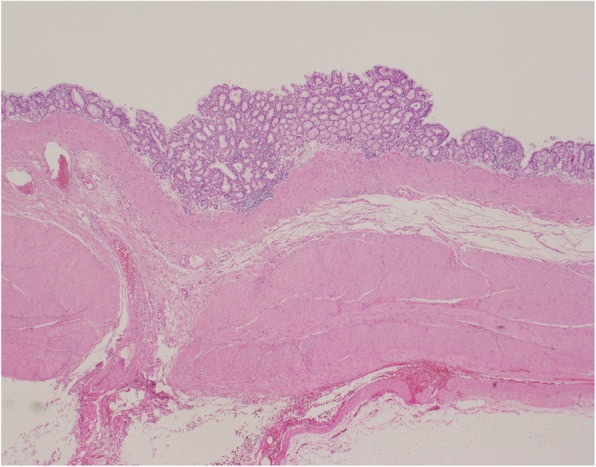
Histopathological study revealing that the mucosa was covered with crypt epithelium, muscularis mucosae, intrinsic muscularis, and serosa and that the tumor wall had a structure very similar to that of the stomach wall

Histopathological study (Fig. 6) revealed that the mucosa was covered with crypt epithelium, muscularis mucosae, intrinsic muscularis, and serosa and that the tumor’s wall had a structure very similar to that of the stomach wall. The mucosa was partially drained by intrinsic gastric glands, but most of them were denucleated. No pancreatic tissue was present, and the tumor had no continuity with the spleen. These findings indicated a diagnosis of GDC that had no continuity with the stomach wall.

The postoperative course was uneventful, and the patient was discharged 6 days after surgery.

The patient’s subsequent clinical course was unremarkable, and she visits our institution on an outpatient basis every 6 months.

## Discussion

GDCs may occur in any anatomical region of the gastrointestinal tract, from mouth to anus, and are present in 1 per 4500–10,000 live births [4]. Gastric duplication is a relatively rare congenital malformation, accounting for approximately 2.9–3.8% of gastrointestinal duplications [5]. Typical GDCs are usually located along the greater gastric curvature, and some others may be located along the anterior or posterior wall of the stomach or in the cardia or pylorus. Most GDCs are single, elliptical, spherical, cystic, and linked with gastric muscular layers.

The essential criteria for diagnosis of GDC are as follows: (1) the whole cyst is contiguous with the stomach wall; (2) the cyst is surrounded by smooth muscle, which is continuous with the muscles of the stomach; and (3) the cyst wall is lined by epithelium of gastric or any other type of gut mucosa [6–8]. More females than males are affected (8:1), and the majority of cases are diagnosed in the pediatric population within the first 3 months of life and rarely after 12 years of age [9, 10].

Because most cases occur along the greater curvature of the stomach, the cysts can potentially compress adjacent organs, such as the pancreas, spleen, and adrenal glands. Therefore, the differential diagnosis includes lesions arising from these organs [11]. It is extremely difficult to preoperatively diagnose GDCs not continuous with the stomach wall, as in our patient; however, because of recent advances in imaging modalities, some informative findings have been reported. The classical radiologic appearance of GDCs on both computed tomography and magnetic resonance imaging (MRI) is of thick-walled cystic lesions with inner lining enhancement and occasional calcifications [11]. MRI can further differentiate the type of the cyst and characterize the cystic contents [12]. The nature of the fluid in the cyst can vary with the presence of bleeding, chronic inflammation, or infection. Therefore, MRI seems to be of less significance than expected in diagnosing GDCs. EUS can be a useful tool for diagnosing GDCs [9, 13]. To obtain further information, endoscopic needle aspiration has been reported to be performed [14]. However, the role of EUS-guided fine-needle aspiration of GDCs is uncertain because the cytological features of GDC may closely resemble those of pancreatic MCNs, and GDCs with elevated levels of carcinoembryonic antigen and carbohydrate antigen 19-9 have been reported, which mimic findings in pancreatic MCNs [7, 15]. In addition, this procedure may cause complications such as hemorrhage and, if the tumor is malignant, peritoneal dissemination of cancer cells.

The cysts may also be manifested by complications such as infection, bleeding, perforation, ulceration, fistula formation, obstruction, and compression [16]. In adults, GDCs are usually asymptomatic and consequently are incidentally diagnosed, as in our patient. However, in some cases, GDCs might manifest with abdominal symptoms such as abdominal mass, pain, and vomiting. In symptomatic cases, surgical resection is often the choice for symptom relief. In asymptomatic cases, surgical resection is controversial. However, in some cases, events such as torsion, perforation, and hemorrhage have been linked to these cysts, and malignant transformation of GDCs, although rare, has been reported [6, 8, 17–19]. Therefore, we believe that resection should be the first treatment option for GDCs.

## Conclusions

GDCs are difficult to differentiate from MCNs until they are laparoscopically resected. Events such as infection, bleeding, perforation, ulceration, fistula formation, obstruction, and compression have been linked to GDCs, and malignant transformation of these cysts has been reported. Therefore, we suggest that resection should be the first treatment option for GDCs.

## Data Availability

The photos used in this case report are published within the report.

## Abbreviations

EUS: Endoscopic ultrasonography
GDC: Gastric duplication cyst
MCN: Mucinous cystic neoplasm
MRI: Magnetic resonance imaging

## References

[1] Wieczorek RL, Seidman I, Ranson JH, Ruoff M. Congenital duplication of the stomach: case report and review of the English literature. Am J Gastroenterol. 1984;79:597–602. 6465107

[2] Perek A, Perek S, Kapan M, Goksoy E. Gastric duplication cyst. Dig Surg. 2000;17:634–6. 10.1159/000051975 11155012

[3] Kim SM, Ha MH, Seo JE, Kim JE, Min BH, Choi MG, *et al.* Gastric duplication cysts in adults: a report of three cases. J Gastric Cancer. 2015;15:58–63. 10.5230/jgc.2015.15.1.58 PMC4389098 25861524

[4] Lee TC, Kim ES, Ferrell LB, Brandt ML, Minifee PK, Midgen C, Domingo RP, Kearney DL. Gastric duplication cysts of the pancreas: clinical presentation and surgical management. Euro J Ped Surg. 2011;21:402–4. 10.1055/s-0031-1279692 21901665

[5] Fox RT, Fowler JT Jr. Duplication of the alimentary tract. J Ped. 1952;41:194–7. 10.1016/s0022-3476(52)80057-5 14955746

[6] Kuraoka K, Nakayama H, Kagawa T, Ichikawa T, Yasui W. Adenocarcinoma arising from a gastric duplication cyst with invasion to the stomach: a case report with literature review. J Clin Pathol. 2004;57:428–31. 10.1136/jcp.2003.013946 PMC1770274 15047751

[7] Johnston J, Wheatley GH 3rd, El Sayed HF, Marsh WB, Ellison EC, Bloomston M. Gastric duplication cysts expressing carcinoembryonic antigen mimicking cystic pancreatic neoplasms in two adults. Am Surg. 2008;74:91–4. 18274440

[8] Horne G, Ming-Lum C, Kirkpatrick AW, Parker RL. High-grade neuroendocrine carcinoma arising in a gastric duplication cyst: a case report with literature review. Int J Surg Pathol. 2007;15:187–91. 10.1177/1066896906295777 17478780

[9] Tanaka M, Akahoshi K, Chijiiwa Y, Sasaki I, Nawata H. Diagnostic value of endoscopic ultrasonography in an unusual case of gastric cyst. Am J Gastroenterol. 1995;90:662–3. 7717335

[10] Takahara T, Torigoe T, Haga H, Yoshida H, Takeshima S, Sano S, Ishii Y, Furuya T, Nakamura E, Ishikawa M. Gastric duplication cyst: evaluation by endoscopic ultrasonography and magnetic resonance imaging. J Gastroenterol. 1996;31:420–4. 10.1007/BF02355033 8726835

[11] Maeda H, Okabayashi T, Nishimori I, Kobayashi M, Morimoto K, Miyaji E, Kohsaki T, Hanazaki K, Onishi S. Diagnostic challenge to distinguish gastric duplication cyst from pancreatic cystic lesions in adult. Intern Med. 2007;46:1101–4. 10.2169/internalmedicine.46.0009 17634707

[12] Hartnick CJ, Barth WH Jr, Cote CJ, Albrecht MA, Grant PE, Geyer JT. Case records of the Massachusetts General Hospital. Case 7-2009: A pregnant woman with a large mass in the fetal oral cavity. N Engl J Med. 2009;360:913–21. 10.1056/NEJMcpc0809061 19246364

[13] Ferrari AP Jr, Van Dam J, Carr-Locke DL. Endoscopic needle aspiration of a gastric duplication cyst. Endoscopy. 1995;27:270–2. 10.1055/s-2007-1005683 7664707

[14] Napolitano V, Pezzullo AM, Zeppa P, Schettino P, D’Armiento M, Palazzo A, Della Pietra C, Napolitano S, Conzo G. Foregut duplication of the stomach diagnosed by endoscopic ultrasound guided fine-needle aspiration cytology: case report and literature review. World J Surg Oncol. 2013;11:33. 10.1186/1477-7819-11-33 PMC3599514 23374143

[15] D’Journo XB, Moutardier V, Turrini O, Guiramand J, Lelong B, Pesenti C, Monges G, Giovannini M, Delpero JR. Gastric duplication in an adult mimicking mucinous cystadenoma of the pancreas. J Clin Pathol. 2004;57:1215–8. 10.1136/jcp.2004.019091 PMC1770488 15509688

[16] Murakami S, Isozaki H, Shou T, Sakai K, Toyota H. Foregut duplication cyst of the stomach with pseudostratified columnar ciliated epithelium. Pathol Int. 2008;58:187–90. 10.1111/j.1440-1827.2007.02209.x 18251783

[17] Mardi K, Kaushal V, Gupta S. Foregut duplication cysts of stomach masquerading as leiomyoma. Ind J Pathol Microl. 2010;53:829–30. 10.4103/0377-4929.72064 21045433

[18] Abdulla MAM, Al Saeed M, Ameer Alshaikh S, Nabar UJ. Adenocarcinoma arising from a gastric duplication cyst: a case report and literature review. Int Med Case Rep J. 2017;10:367–72. 10.2147/IMCRJ.S138616 PMC5683793 29158693

[19] Coit DG, Mies C. Adenocarcinoma arising within a gastric duplication cyst. J Surg Oncol. 1992;50:274–7. 10.1002/jso.2930500417 1640716

